# Amyloidosis awareness and colchicine adherence in patients with familial Mediterranean fever: a cross-sectional study in an endemic region

**DOI:** 10.1007/s10067-026-08221-9

**Published:** 2026-06-12

**Authors:** Elif Dincses-Nas, Sevilay Batibay, Tugce Bozkurt, Ender Igneci, Seda Gunay Akyol, Esen Kasapoglu

**Affiliations:** Department of Internal Medicine, Division of Rheumatology, Istanbul Medeniyet University Goztepe Prof. Dr. Suleyman Yalcin City Hospital, Kadikoy Istanbul, Turkey

**Keywords:** Amyloidosis, Awareness, Colchicine, Familial Mediterranean fever, Medication adherence, Surveys and questionnaires

## Abstract

**Introduction:**

Familial Mediterranean Fever (FMF) is the most common hereditary autoinflammatory disease, and secondary amyloidosis remains its most serious complication. We aimed to investigate amyloidosis awareness, colchicine adherence and related factors among FMF patients.

**Methods:**

In this cross-sectional single-center study, patients with FMF completed a face-to-face questionnaire assessing amyloidosis awareness using structured questions and colchicine adherence through self-reported medication use. Demographic and clinical data were obtained from medical records. Factors associated with amyloidosis awareness and colchicine adherence were evaluated using univariate and multivariable analyses.

**Results:**

A total of 185 patients were included; 64% were female. The median age was 38 years and disease duration was 13 years. Regular colchicine use was reported by 144 (78%) patients and was associated with awareness of its importance, older age, higher colchicine dose, and fewer attacks. Only 51 (27%) patients were completely or partially aware of amyloidosis, whereas 153 (83%) were aware of the risk of renal failure. Amyloidosis awareness was associated with younger age and higher education level. In multivariable analysis, regular colchicine use was independently associated with fewer attacks and a higher daily colchicine dose; while amyloidosis awareness was independently associated with awareness of the amyloidosis risk related to irregular colchicine use.

**Conclusion:**

Awareness of amyloidosis remains insufficient among patients with FMF, although colchicine adherence was high. Targeted educational strategies may be needed to improve awareness of amyloidosis and its prevention.

**Table:** 

**Key Points** • *Awareness of amyloidosis was limited (27%) and was associated only with awareness of the amyloidosis risk related to irregular colchicine use*. • *Treatment adherence is closely linked to higher colchicine dose and fewer attacks*.

**Supplementary Information:**

The online version contains supplementary material available at 10.1007/s10067-026-08221-9.

## Introduction

Familial Mediterranean Fever (FMF) is the most common autoinflammatory disease and predominantly affects the populations originating East Mediterranean basin, notably Turks, non-Ashkenazi Jews, Armenians, and Arabs [1, 2]. The “MEFV" (MEditerranean FeVer) gene that is located on the short arm of chromosome 16, encodes a protein termed pyrin; and is associated with FMF. Typical clinical manifestations of FMF are self-limited, recurrent febrile serositis and/or arthritis attacks. Patients have generally attack-free intervals, but subclinical inflammation might continue during these intervals. The most important complication of FMF is AA (secondary) amyloidosis which is a result of uncontrolled inflammation. It leads to significant morbidity and mortality due to end-stage renal disease and other visceral organ manifestations.

The prevalence of FMF in Turkey from the latest study using national electronic health records; was reported to be 139/10000 [3]. This study which used ICD 10 coding rather than classification criteria, reported a much higher rate than the previous prevalence of 1–8/1000 in earlier studies [4–6]. Parallel to this high prevalence in our country, the most common cause (78%) of AA amyloidosis is FMF [7]. Although the prevalence of amyloidosis in FMF was higher in previous years, a study conducted in 2005 with the largest patient cohort from Turkey, reported declining rate of biopsy-proven amyloidosis in 12.9% of FMF patients [8]. More recent studies in 2012 and 2013 reported this rate to be lower at 6.3% and 8.6% respectively [9, 10]. In the most recent 2 studies conducted over the past two years, this frequency has similarly been reported as 10% [11, 12].

Colchicine is the main treatment for decreasing the frequency and severity of FMF attacks, and is also proven to prevent amyloidosis when used in optimal dose and regularly [13–16]. Therefore, the declining rate of AA amyloidosis in recent years is explained with an earlier initiation of colchicine at the time of FMF diagnosis. The risk of AA amyloidosis has not disappeared completely with the improvement in the treatment. Before starting anti interleukin-1 therapy in the active disease, assessing drug adherence and confirmation of true colchicine resistance is essential. Poor adherence to colchicine may mimic treatment resistance and contribute to poor disease control and complications. Despite being such an important complication, as far as we know, there is no study investigating the awareness of amyloidosis among patients with FMF. One of the main goals in the management of FMF is to prevent the development of amyloidosis. While rheumatologists aim to achieve this, patients’ awareness of amyloidosis and their active participation in this process may improve treatment success.

The primary aim of this study was to determine amyloidosis awareness and its associated factors. In addition, since inadequate dosing and non-adherence are among the risk factors for amyloidosis, we hypothesized that patients who are aware of amyloidosis may have better medication adherence and more optimal colchicine use. Accordingly, our secondary aim was to assess colchicine adherence and its relationship with amyloidosis awareness.

## Materials and methods

### Study design and population

In our single-center cross-sectional study; we included 185 patients, aged ≥ 18 years with a diagnosis of FMF according to the Tel-Hashomer Criteria [17]. The patients were consecutively enrolled from the Rheumatology Outpatient Clinic of Goztepe Prof Dr Suleyman Yalcin City Hospital (Istanbul, Turkey) between April 2025 and February 2026. Exclusion criteria were as follows: patients with cognitive dysfunction, those diagnosed less than 6 months earlier, and those who had fewer than two follow-up visits after diagnosis.

### Data collection and definitions

The demographic characteristics (age, sex, level of formal education) and clinical characteristics (age at disease onset, age at diagnosis, disease duration, current medications, presence of concomitant diseases, MEFV mutations, number of attacks in the last year, presence of biopsy-proven amyloidosis, family history of FMF and amyloidosis) were recorded from patient files.

All patients completed a face-to-face questionnaire including three domains addressing: Colchicine use (Q1-4), follow up (Q5-6) and awareness of amyloidosis (Q7-13).

In the first domain, a structured questionnaire-based approach focusing on patients’ knowledge about importance/effects of colchicine, self-reported medication-taking behaviors and barriers/reasons for non-adherence was employed. Colchicine adherence was categorized as follows: “very regular use” was defined as taking more than 80% of the prescribed doses, “irregular use” as missing more than half of the doses, and “partially regular use” as an adherence level between these two categories. In the second domain, rheumatology follow-up visits and disease-related laboratory tests were asked. In the third domain; amyloidosis awareness was assessed by a rheumatologist using two sequential questions: whether the patient had heard of amyloidosis and how they defined it. Patients who defined amyloidosis as “organ dysfunction due to accumulation secondary to persistent inflammation” were considered to have “complete awareness,” whereas those providing a general statement such as “it can cause harm if the disease is severe or uncontrolled” were classified as having “partial awareness”. The other relevant questions were about the association between the risk of amyloidosis and irregular colchicine use and also awareness of renal complications (chronic kidney disease and proteinuria). The survey was reported according to published recommendations for survey research methodology [18].

The full survey questionnaire is provided in the Supplementary Material.

At the end of the questionnaire, a brief written information sheet about amyloidosis and its prevention was provided to each patient. Informed consents were obtained from all patients who participated in this study.

The study was conducted in accordance with the ethical standards outlined in the 1964 Declaration of Helsinki and its later amendments. Ethical approval was obtained from the Istanbul Medeniyet University Ethics Committee (2025/05–08).

### Statistical analysis

The Kolmogorov–Smirnov normality test was used for the distribution pattern of the variables. Continuous data are presented as mean and standard deviation (SD) or median and interquartile range (IQR). Categorical variables are presented as absolute numbers and percentages (%). Chi-square test or Fisher’s exact test were used for the comparison of categorical variables, and Mann–Whitney U test was used for comparison of continuous variables between two groups. A two-tailed *P*-value < 0.05 was considered statistically significant. For the multivariate analysis, the possible factors identified with the univariate analyses were further entered into the logistic regression analysis to determine independent predictors of outcomes. Hosmer- Lemeshow goodness of fit statistics were used to assess model fit. All tests were performed using IBM SPSS Statistics 24 (SPSS Inc, Chicago, IL, USA).

## Results

### Clinical and demographic characteristics of the patients (Table 1)

**Table 1:** Demographic and clinical characteristics of the patients

	Total *n* = 185
Female *n* (%)	119 (64)
Median age (IQR)	38 (26–49)
Education level ≥ high school, *n* (%)	129 (70)
Median age at disease onset (years) (IQR)	10 (7–18)
Median duration of FMF diagnosis (years) (IQR)	13 (6.5–20)
Median number of attacks during last year (IQR)	2 (0–5)
Having* at least one pathogenic exon 10 variant** *n* (%)	118 (90)
Having 1^st^ or 2^nd^ degree relative with FMF diagnosis *n* (%)	90 (49)
Comorbid inflammatory disease *n* (%)	17 (9)
Median colchicine dose mg/day (IQR)	1.5 (1–2)
Patients using anti–IL-1 therapy, n (%)	13 (7)
Patients with amyloidosis diagnosis *n* (%)	7 (4)

Anti IL-1: Anti interleukin-1, FMF: Familial Mediterranean Fever

*: Available in 131 patients, **: M694V, M680I, V726A, M694I

A total of 185 FMF patients were included, of whom 119 (64%) were female. The median age of patients was 38 (IQR:26–49). The educational status was high school or higher level in 129 (70%), primary school in 32 (17%) and 3 (2%) had no formal education. The median age at symptom onset was 10 years (IQR:7–18), median disease duration was 13 (IQR:6.5–20) years and diagnostic delay was median 6 (IQR:1–16) years. Seventeen (9%) patients had other inflammatory comorbidities (13 spondyloarthritis, 1 Crohn’s disease, 2 rheumatoid arthritis and 1 Behçet’s disease). Forty-six (25%) of patients had at least a first degree relative with a diagnosis of FMF. Fifty-seven (31%) of the patients did not have any relatives diagnosed with FMF. MEFV genetic test results were present in 131 (71%) patients; 90% had at least one pathogenic exon 10 variant, the most common pathogenic variant was M694V in 93 (71%) patients and 38 patients (29%) were homozygous; 9 (7%) had E148Q or R202Q variants. Only 4 patients did not have any MEFV variants. The median colchicine dose was 1.5 (IQR:1–2) mg/day. Anti interleukin-1(IL-1) treatment was given to 13 (7%) patients and biopsy proven secondary amyloidosis was present in 7 (4%) patients.

### Comparison of patients according to colchicine adherence (Table 2)

**Table 2:** Comparison of patients according to colchicine adherence

	Very regular colchicine use (≥ 80% of doses) ***n*** = 144	Not very regular colchicine use (< 80% of doses) ***n*** = 41	***p*** value
Female sex, *n* (%)	93 (65)	26 (63)	0.89
Median age (IQR)	38.5 (26–50)	30 (22–40)	**0.008**
Education level ≥ high school, *n* (%)	97 (68)	32 (80)	0.13
M694V homozygous mutation *n* (%)	27 (19)	10 (24)	0.42
Median duration of FMF diagnosis (years) (IQR)	13 (6–20)	15 (7–22)	0.24
Median number of attacks during the last year (IQR)	2 (0–4)	3 (2–6.5)	**0.001**
Knowledge of the importance/effect of colchicine *n* (%)	94 (65)	19 (46)	**0.028**
Awareness about the risk of amyloidosis in treatment non adherence	40 (28)	9 (22)	0.45
Awareness about the risk of “renal failure” *n* (%)	117 (81)	36 (88)	0.32
Regular rheumatology follow-up, *n* (%) *	94 (65)	24 (58)	0.42
Median drug dose mg/d (IQR)	1.5 (1–2)	1 (1–1.5)	**0.050**
“Compressed” colchicine preparation use	34 (23.6)	4 (9.7)	0.076
Patients using anti–IL-1 therapy, *n* (%)	10 (7)	3 (7)	1.0
Patients with amyloidosis diagnosis *n* (%)	6 (4)	1 (2)	1.0

*At least every 6 months

Anti IL-1: Anti interleukin-1, CKD: Chronic Kidney Disease, FMF: Familial Mediterranean Fever

The knowledge about the effects and/or importance of colchicine was 113 (61%), despite this 144 (78%) of patients reported using colchicine very regularly. The main reason (71%) for non-adherence was forgetfulness (“unintentional non-adherence”) and further 5 (12%) was omission of doses because of their rare/mild attacks. Adverse events were reported as the reason for non-adherence only in the 4 of 41 (10%) patients. Regular colchicine use was significantly associated with the knowledge about the effects and/or importance of colchicine, older age and less median number of attacks (*p* = 0.028, *p* = 0.008, *p* = 0.001 respectively). Also, the median drug dose was higher in patients with very regular colchicine use (1.5 mg/d vs 1 mg/d, *p* = 0.05). In multivariable logistic regression analysis, a higher number of attacks in the last year was associated with a lower likelihood of regular colchicine use (OR: 0.81, 95% CI: 0.72–0.91, *p* < 0.001), whereas higher daily colchicine dose was associated with an increased likelihood of regular use (OR: 3.00, 95% CI: 1.12–8.07, *p* = 0.030).

### Comparison of patients with and without amyloidosis awareness (Table 3)

**Table 3:** Comparison of patients with and without amyloidosis awareness

	Patients with Amyloidosis Awareness *n* = 51	Patients without Amyloidosis Awareness *n* = 134	*p* value
Female sex, *n* (%)	36 (71)	83 (62)	0.27
Median age (IQR)	31 (25–45)	39.5 (26–49)	**0.020**
Education level ≥ high school, *n* (%)	43 (84)	86 (65)	**0.011**
Median duration of FMF diagnosis (years) (IQR)	13 (6–19)	13 (6.7–21.2)	0.54
M694V homozygous mutation *n* (%)	9 (18)	28 (21)	0.24
Median number of attacks during the last year (IQR)	3 (1–6)	2 (0–5)	0.19
Aware of the risk of amyloidosis associated with irregular use of colchicine *n* (%)	43 (84)	6 (4)	** < 0.001**
Very regular colchicine use, *n* (%)	42 (82)	102 (76)	0.36
Median drug dose mg/d (IQR)	1.5 (1–2)	1.5 (1–2)	0.27
Regular rheumatology follow-up, *n* (%) *	38 (74)	80 (60)	0.06
More than 2 different rheumatologists ever visited *n* (%)	35 (67)	87 (65)	0.22
First degree family history of FMF, *n* (%)	11 (21)	35 (26)	0.52
Patients using anti–IL-1 therapy, *n* (%)	7 (14)	6 (4)	**0.028**
Awareness about the risk of “renal failure” *n* (%)	50 (98)	103 (77)	**0.001**

Anti IL-1: Anti interleukin-1, FMF: Familial Mediterranean Fever

*: At least every 6 months

Seventy-three (39.5%) of patients had heard the term of amyloidosis, however 27 (37%) of them had no idea about its meaning, while 24 (33.8%) of them know the meaning of amyloidosis satisfactorily and 22 (30.1%) knows it partially. Apart from this; 5 patients knew amyloidosis partially even though they had not heard the specific term. A total of 51 (27%) patients had at least partial knowledge of the meaning of amyloidosis which were classified as “patients with awareness”. Only 20 (40%) of aware patients learned it from their rheumatologists; while 15 (30%) learned it from their relatives or own research through the internet/social media. Essentially, 153 (83%) of all patients were aware of the risk of chronic kidney disease, moreover 107 (58%) patients know the risk of proteinuria when they were asked specifically. The median age of patients with amyloidosis awareness was significantly younger (31 vs 39.5 years, *p* = 0.02) with significantly higher educational level (high school and/or university) (84% vs 65%, *p* = 0.011). Using anti IL-1theraphy was significantly associated with amyloidosis awareness (14% vs 4%, *p* = 0.028). Also, awareness about the risk of “renal failure” was significantly associated with amyloidosis awareness as expected (98% vs 77%, *p* = 0.001). Among patients who were aware of amyloidosis, 37% (16/43) of those with a higher level of education had learned about it through their own research and 60% (26/43) of them had heard from their rheumatologists or other physicians. In contrast, all patients with lower level of education who were aware of amyloidosis (*n* = 8) had learned about it from rheumatologists or other physicians rather than through personal research. Patients with amyloidosis awareness demonstrated a significantly greater understanding of the association between irregular colchicine compliance and amyloidosis development compared to patients without such awareness (*p* < 0.001).

Awareness of renal failure risk was found to be significantly associated only with longer disease duration (median 7.5 vs 14 years, *p* = 0.022). However, in multivariable logistic regression analysis, amyloidosis awareness was associated only with increased awareness of the risk of amyloidosis related to irregular colchicine use (OR: 0.009, 95% CI: 0.003–0.032, *p* < 0.001).

## Discussion

In our study only 27% of FMF patients knew the meaning of amyloidosis completely or at least partially, whereas 83% were aware of the risk of chronic kidney disease particularly. Looking at these rates, renal complication was seen to be well-known; yet amyloidosis is a systemic disease and the awareness of amyloidosis was found to be low.

This finding observed in our study may have several explanations: During patient education physicians may have emphasized the renal complications more without specifically using the term “amyloidosis.” Alternatively, patients may have forgotten or underestimated the information provided at the time of diagnosis or at the initiation of treatment. Another possible explanation for insufficient or incomplete patient education by healthcare providers may be limited time, insufficient per patient examination duration. The association between amyloidosis awareness and younger age, as well as higher educational level, may be explained by the greater tendency of these patients to seek information independently through personal research.

Awareness of complications remains a persistent challenge in chronic diseases. In a 2002 study investigating one of the most common chronic diseases, diabetes mellitus, awareness of its most fatal complication—cardiovascular disease (CVD)—was notably low, with only 32% of patients recognizing CVD as a serious complication [19]. Although limited access to online health information at that time may partly explain this finding, the authors primarily attributed it to insufficient physician–patient communication and emphasized the need to improve it.

In our study, awareness of renal complications in FMF appears higher in comparison; however, greater focus should be placed on modifiable risk factors of amyloidosis.

Interestingly, awareness of amyloidosis—or even awareness of renal failure risk—was not significantly associated with colchicine adherence. These findings suggest that poor adherence is not solely attributable to insufficient disease knowledge and support the observation that forgetfulness is the predominant reason for irregular drug use. The higher awareness among younger patients may be related to greater internet use or learning from social media and/or online sources. Unfortunately, awareness of amyloidosis doesn’t seem to affect the young patients’ attitudes directly on treatment adherence. This finding is similar with a study which aimed to develop a “treatment adherence scale for rheumatic diseases” and better drug adherence can be attributed to older adults’ more experience in managing chronic diseases and/or an increased sense of responsibility for daily drug use [20]. Drug adherence is one of the modifiable risk factors associated with increased risk for amyloidosis in FMF patients [21]. In our study, although it was not significant in multivariate analysis, patients who knew the importance of regular colchicine, tend to have better drug adherence.

So, we suggest that repeated reminders and reinforcement during clinical visits may be beneficial. Particularly, since non-adherent patients reported to attend routine follow-up visits at similar frequencies as the adherent patients; clear and repeated explanations of the importance and benefits of colchicine use may help improve treatment adherence.

There are previous studies evaluating colchicine adherence which used standardized and validated tools or electronic data rather than simple self-report. In the first study from Turkey, 66.5% of patients declared regular use to investigators however adherence assessed by the “Compliance Questionnaire on Rheumatology” was 16%, which was surprisingly lower [22]. This highlights a discrepancy between self-reported and objectively measured adherence. Similarly in an earlier report (both adult and pediatric, total *n* = 38 patients) only 13% of the patients filled all the colchicine prescriptions they received from their physician and 34% filled less than 50% of the prescriptions they got that year [23]. These findings show that more than 40% of the patients actually had poor compliance. So, reliance on only self-reported measures about adherence introduces the risk of response bias in these kinds of study designs. Another very recent study from Turkey using the specific “Medication Adherence Scale for FMF (MASIF)” reported a relatively high adherence rate of 75% which was similar to our result [24, 25]. In that study illness perception was also investigated and patients with clearer disease understanding demonstrated better adherence. In our study, regular colchicine use was reported as 78% based solely on patient questioning, however awareness of an important complication was not associated with better adherence. Two previous studies from Turkey, both including smaller patient populations, evaluated colchicine adherence using a simple self-reported assessment of whether patients were taking colchicine treatment and reported adherence rates of 64.5% and 89% [26, 27]. However, these adherence rates were not consistently reflected by improved clinical outcomes or lower annual attack frequencies. In contrast, we observed a significant association between regular colchicine use and a lower number of attacks, supporting the clinical relevance of our adherence assessment.

This study has several limitations. It was conducted in a single tertiary rheumatology outpatient clinic in Istanbul, rather than being a multi-center study which could be potentially limiting the generalizability. Also, the proportion of female patients was slightly higher than expected compared with the classical distribution of FMF. A validated tool for assessing medication adherence was not used, however our primary aim was not to quantify adherence in detail but to explore the underlying reasons for non-adherence. Therefore, a structured questionnaire-based approach focusing on patients’ self-reported medication-taking behavior and barriers was employed. We found that colchicine adherence was significantly associated with a lower number of attacks and a higher daily dose, which may support the internal consistency of questionnaire responses regarding medication use. Also, considering the potential lack of standardization in assessing amyloidosis knowledge, we did not accept merely having heard the term “amyloidosis” as sufficient awareness. Beyond recognizing the term, we verified patients’ knowledge by asking them to define it. Although we initially categorized patients separately in the questionnaire as those providing “complete” and “partial” answers, we considered that anxiety or recall difficulties might affect their answers. Therefore, for the main analyses, we grouped patients as “unaware” and “at least partially aware.” In addition, through two separate questions, we assessed patients’ ability to correctly identify the association between amyloidosis risk and irregular colchicine use, as well as its consequence of renal failure. These responses were correlated with overall amyloidosis knowledge, which may indirectly support the internal consistency of our assessment.

One of the strengths of the study is; to our knowledge this is the first study evaluating complication awareness in patients from an endemic country for FMF. Importantly, patients were not only asked whether they had heard the term “amyloidosis,” but also about their understanding of its meaning and about the most clinically significant complications, specifically renal failure and proteinuria. Also, we investigated the potential modifiable parameters which can affect treatment adherence. At the end of the study, written information was provided to patients in order to address identified knowledge gaps about the importance of regular colchicine use for optimal control of inflammation and so prevention from amyloidosis.

## Conclusion

Our findings highlight a substantial gap between awareness of renal complications and true understanding of amyloidosis in FMF patients. While knowledge of amyloidosis alone does not appear sufficient to improve treatment adherence, targeted, repeated, and structured patient education—particularly emphasizing the importance of regular colchicine use—may help address modifiable risk factors for AA amyloidosis.

## Supplementary Information

Below is the link to the electronic supplementary material.

Supplementary file1 (DOCX 16 KB)

## Data Availability

The data of this study is available from the corresponding author upon request.
