# Supplementary material for: Amyloidosis awareness and colchicine adherence in patients with familial Mediterranean fever: a cross-sectional study in an endemic region
Source: Clin Rheumatol. 2026 Jun 12;45(7):4467–73. doi: 10.1007/s10067-026-08221-9 (PMC13342140; doi:10.1007/s10067-026-08221-9)
Supplement: Supplementary file 1 — Supplementary file1 (DOCX 16 KB) [file 10067_2026_8221_MOESM1_ESM.docx]

**AWARENESS OF AMYLOIDOSIS IN PATIENTS WITH FAMILIAL MEDITERRANEAN FEVER (FMF)**

Date:
Patient ID:

Colchicine use:

1. Does the patient know correctly why they are using colchicine?
□ No = 0 □ Yes = 1 (decreasing the frequency and severity of febrile attacks, preventing inflammation)

2. How do you use colchicine?
Regular (taking full dose ≥80%) = 1
Partially regular (50-80%) = 2
Irregular (<50% adherence / frequent missed doses) = 3

3. If not using regularly, what is the reason?
I forget = 1
Cannot obtain/prescribe the drug = 2
I experience side effects = 3
I do not feel the need due to infrequent attacks = 4
Other = 5

4. Have you experienced side effects related to colchicine?
□ No = 0 □ Yes:
Diarrhea = 1 / Elevated liver enzymes = 2 / Elevated CK = 3 / Other = 4

Follow-up:

5. Do you attend rheumatology follow-up at least twice a year (every 6 months) (for at least the last 3 years)?
□ No = 0 □ Yes = 1

6. Do you have recommended tests (blood/urine) performed at least twice a year (for at least the last 3 years)?
□ No = 0 □ Yes = 1

Awareness of amyloidosis:

7. Have you ever heard the term “amyloidosis”?
□ No = 0 □ Yes = 1

8. Do you know its meaning correctly?
□ No = 0 □ Yes (fully) = 1 (organ dysfunction/damage due to accumulation secondary to persistent inflammation) □ Partially = 2 (it can cause harm if the disease is severe or uncontrolled)

9. Did you know that not using colchicine may increase the risk of developing amyloidosis?
□ No = 0 □ Yes = 1

10. Did you know that not using colchicine or using it irregularly may increase the risk of kidney failure?
□ No = 0 □ Yes = 1

11. Did you know that not using colchicine or using it irregularly may develop proteinuria?
□ No = 0 □ Yes = 1

12. From whom or where did you learn the term “amyloidosis”?
From a rheumatologist = 1  From another physician = 2
From family members = 3   From a friend = 5
Self-research (internet) = 4

13. How many different rheumatologists have evaluated you so far?
1 / 2 / More
